# Predictors for patients understanding reason for hospitalization

**DOI:** 10.1371/journal.pone.0196479

**Published:** 2018-04-27

**Authors:** Himali Weerahandi, Boback Ziaeian, Robert L. Fogerty, Grace Y. Jenq, Leora I. Horwitz

**Affiliations:** 1 Department of Medicine, NYU School of Medicine, New York, NY, United States of America; 2 Department of Population Health, NYU School of Medicine, New York, New York, United States of America; 3 Division of Cardiology, UCLA Medical Center, Los Angeles, CA, United States of America; 4 Division of Cardiology, Veteran Affairs Greater Los Angeles Healthcare System, Los Angeles, CA, United States of America; 5 Division of General Internal Medicine, Yale School of Medicine, New Haven, CT, United States of America; 6 Division of Geriatrics and Palliative Medicine, University of Michigan, Ann Arbor, MI, United States of America; 7 Center for Healthcare Innovation and Delivery Science, NYU Langone Medical Center, New York, New York, United States of America; Public Library of Science, UNITED KINGDOM

## Abstract

**Objective:**

To examine predictors for understanding reason for hospitalization.

**Methods:**

This was a retrospective analysis of a prospective, observational cohort study of patients 65 years or older admitted for acute coronary syndrome, heart failure, or pneumonia and discharged home.

Primary outcome was complete understanding of diagnosis, based on post-discharge patient interview. Predictors assessed were the following: jargon on discharge instructions, type of medical team, whether outpatient provider knew if the patient was admitted, and whether the patient reported more than one day notice before discharge.

**Results:**

Among 377 patients, 59.8% of patients completely understood their diagnosis. Bivariate analyses demonstrated that outpatient provider being aware of admission and having more than a day notice prior to discharge were not associated with patient understanding diagnosis. Presence of jargon was not associated with increased likelihood of understanding in a multivariable analysis. Patients on housestaff and cardiology teams were more likely to understand diagnosis compared to non-teaching teams (OR 2.45, 95% CI 1.30–4.61, p<0.01 and OR 3.83, 95% CI 1.92–7.63, p<0.01, respectively).

**Conclusions:**

Non-teaching team patients were less likely to understand their diagnosis. Further investigation of how provider-patient interaction differs among teams may aid in development of tools to improve hospital to community transitions.

## Introduction

Almost one in five Medicare beneficiaries are readmitted within 30 days of hospital discharge, making the transition from hospital back to community a particularly vulnerable time for patients.[1] Inpatient discharge typically occurs when patients have improved sufficiently to no longer require inpatient care. However, at this point the acute illness typically is not completely resolved and patients may still need additional care in the immediate post-discharge period. Patient education, including the reason for admission, helps prepare the patient for such post-discharge care requirements. Understanding diagnosis is an important part of framing a treatment and self-monitoring plan to a patient, and previous studies have demonstrated that patients’ understanding of their treatment plan impacts self-management behaviors.[2] Poor understanding of the reason for admission may negatively affect patients’ ability to understand discharge instructions and their importance, and may impede patients from successfully carrying out discharge instructions. Thus, ensuring patients understand their diagnosis is an important area to target to reduce readmissions.[3]

We recently demonstrated that only about 60% of patients were able to accurately describe their diagnosis in post-discharge interviews,[4] and the majority of patients do not understand medication changes on discharge [5], which is consistent with prior studies.[6] The reasons for this failure, however, are uncertain. In order to effectively intervene in improving patient understanding of diagnosis and other post-discharge care requirements, it is essential to identify systems-level, modifiable factors that affect patient comprehension.

To examine systems-level contributors to patient understanding, we examined data from the DIagnosing Systemic failures, Complexities and HARm in GEriatric discharges study (DISCHARGE).

## Methods

### Study cohort

This study is a retrospective chart review of data collected from DISCHARGE. DISCHARGE was a prospective, observational cohort study of patients 65 years or older admitted to a single academic medical center for acute coronary syndrome, heart failure, or pneumonia between May 2009 and April 2010 who were subsequently discharged to home.[4,5,7] Additional eligibility criteria included speaking English or Spanish, not being in hospice care, and participating in a telephone interview; caregivers could also take part on behalf of patients. Patients were excluded if they appeared delirious or failed the Mini-Cog mental status screen while admitted (defined as a score <3).[8] DISCHARGE was approved by the Yale's Human Investigation Committee. Verbal informed consent was obtained from all study participants to participate in a telephone interview and included separate permission to the investigators to review their medical records. Verbal informed consent was approved by the ethics committee at Yale. Data was de-identified prior to data analysis by non-Yale collaborators.

The DISCHARGE study included a telephone interview with patients or their caregivers within one week of discharge. The interview included questions about the reason for admission and the amount of notice patients had prior to discharge. This interview was conducted by trained, non-clinical personnel. A medical record review was also conducted by experienced nurse abstractors.

### Measures

Complete understanding of diagnosis was the primary outcome. Verified patient understanding of reason for hospitalization was performed by comparing patients’ responses to the question “please tell me the reason you were in the hospital” with administrative billing data of principal diagnosis, the wording in the diagnosis section of the discharge instructions, and investigators’ assignment of the patient to the heart failure, pneumonia, or acute coronary syndrome cohort. If the patient had several main diagnoses, description of any of them was defined as complete understanding. Patient understanding of discharge diagnosis was considered to be complete if the patient’s language would make it clear to a medical professional what the diagnosis for the hospitalization was, even without technical language. For example, if a patient had been admitted for heart failure, we gave full credit for understanding both for responses such as “heart failure” and for “fluid in the legs” or “weak heart.” We gave partial credit for provision only of symptoms consistent with the diagnosis (such as “trouble breathing” for heart failure), and no credit for vague symptoms (“weakness,” “not feeling well”) or lack of knowledge (“don’t know,” “my doctor told me to come in”). For this study we dichotomized understanding as complete or not complete.

Modifiable systems predictors assessed for complete understanding of diagnosis were the presence of jargon on patient discharge instructions, type of medical team, whether outpatient provider knew if the patient was admitted, and whether the patient had more than more than one day notice prior to discharge. Non-modifiable predictors were age, race, sex, diagnosis, education, and income.

To define jargon on discharge instructions, every reason for hospitalization was recorded and categorized by study investigators. Disagreements were resolved through iterative discussion. Intelligible language was defined by any term that was commonly used in spoken English (e.g., pneumonia or heart attack), any medical jargon for chronic diseases that the investigators believed that patients would likely be familiar with (e.g., congestive heart failure or atrial fibrillation), and any medical jargon for acute events that was commonly used by patients in the study to describe their hospitalization (e.g., catheterization). Remaining terms were defined as medical jargon (S1 Appendix). Type of medical team (non-teaching, housestaff, or cardiology) was abstracted from the chart. At this institution, non-teaching teams are composed of a hospitalist working with either a physician assistant or a nurse practitioner. Housestaff teams are composed of medical students, interns, residents, and attending physicians. The attending on the housestaff team may be either a hospitalist or primarily outpatient faculty physician. Cardiology teams are composed of a cardiologist and either housestaff, a nurse practitioner, or a physician assistant. Data on whether the outpatient provider knew if the patient was admitted and whether the patient had more than one day notice prior to discharge was obtained from the post-discharge interview. Data on age, race, and length of stay were obtained from hospital administrative databases.

### Statistical analysis

The characteristics of the study population were summarized with descriptive analyses. We summarized dichotomous variables as proportions and continuous variables with a mean and standard deviation if they were normally distributed, if not, with a median and interquartile range. We examined the relationship of systems predictors to fully understanding/not fully understanding hospital diagnosis using a multivariable logistic regression model controlling for age, race, sex, diagnosis, education, and income. Data were analyzed using SAS, version 9.3 (SAS Institute, Cary, NC).

## Results

### Sample characteristics

**Fig 1:**
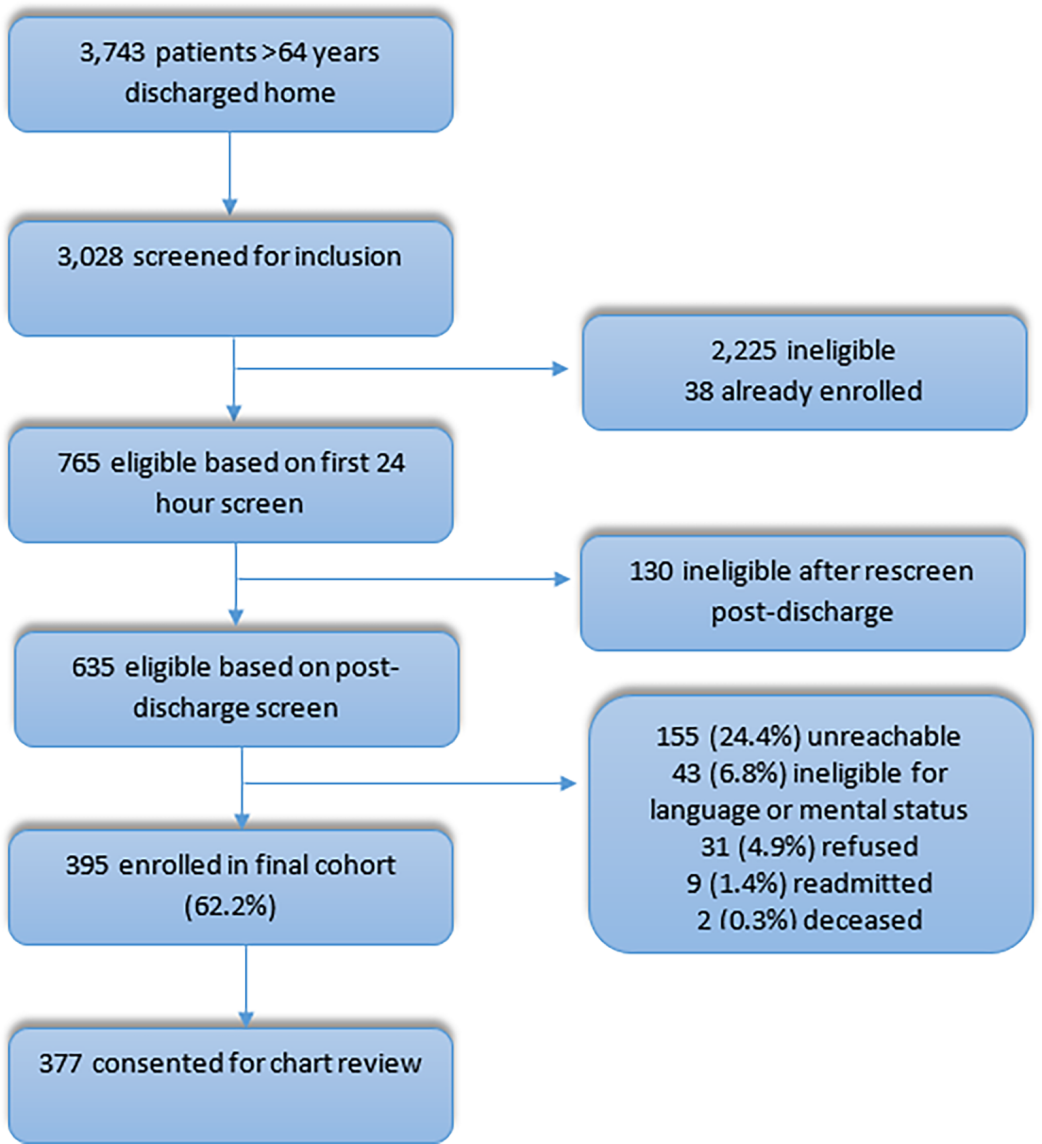
Flow diagram of enrolled patients.

A total of 3743 patients over 64 years old were discharged home from the medical service at our medical center during the study period, and 3028 patients were screened for eligibility within 24 hours of admission. Screening identified 635 eligible admissions and we enrolled 395 patients (62.2%) in the study. Of these, 377 provided permission for chart review and there was data on understanding diagnosis on 374 patients (Fig 1).

In this group, 222 (59.8%) patients completely understood the diagnosis. The mean age in both groups was about 77, and sex distribution was similar between the two groups (Table 1). Patients who did not completely understand their diagnoses were more likely to have been admitted for HF (50.7% vs 30.2%, p<0.01), have less formal education (p<0.01), and have a lower income (p<0.01).

**Table 1:** Baseline characteristics of the study cohort (n = 374).

Characteristic	Completely understood diagnosis (n = 222)	Did not completely understand diagnosis (n = 152)	p-value
**Condition (n = 374)**			
Acute Coronary Syndrome	125 (56.3%)	70 (46.1%)	0.05
Community-Acquired pneumonia	54 (24.3%)	36 (23.7%)	0.89
Heart Failure	67 (30.2%)	77 (50.7%)	<0.01
**Age, mean (SD) (n = 366)**	76.9 (7.6)	77.0 (7.5)	0.98
**Male sex (n = 366)**	123 (56.2%)	78 (53.1%)	0.56
**English-speaking (n = 366)**	212 (98.2%)	143 (97.3%)	0.47
**Race/ethnicity (n = 369)**			0.16
Non-Hispanic white	188 (85.8%)	116 (78.9%)	
Non-Hispanic black	18 (8.2%)	23 (15.7%)	
Hispanic	8 (3.7%)	7 (4.8%)	
Other	5 (2.3%)	1 (0.7%)	
**Education (n = 365)**			<0.01
<9th grade	19 (8.8%)	21 (14.5%)	
9th-12th grade	34 (15.7%)	22 (15.2%)	
High school diploma or GED	51 (23.5%)	53 (36.6%)	
College degree	78 (35.9%)	28 (19.3%)	
Graduate degree	35 (16.1%)	21 (14.5%)	
**Yearly Income (n = 340)**			<0.01
0-$18,000	48 (23.5%)	51 (37.5%)	
$18,000-$30,000	25 (12.3%)	26 (19.1%)	
$30,000-$45,000	23 (11.3%)	8 (5.9%)	
$45,000-$65,000	19 (9.3%)	5 (3.7%)	
>$65,000	50 (24.5%)	20 (14.7%)	
No response	39 (19.1%)	26 (19.1%)	

### Predictors for understanding diagnosis

In this cohort, 367 patients had a diagnosis written on their discharge instructions, which was coded for the presence of jargon. A bivariate analysis demonstrated that patients with jargon on their discharge instructions were more likely to completely understand their diagnosis compared with patients without jargon on their discharge instructions (70.5% vs 56.3% respectively, p = 0.01, see Table 2). As this was an unexpected finding, we performed a stratified analysis by diagnosis to examine whether this relationship was disease specific. Stratified analysis by diagnosis demonstrated that HF patients with jargon were more likely to understand their diagnosis (66.7% vs 43.5%, p = 0.05). Most of these patients (57%) had “coronary artery disease/unstable angina” listed on their discharge instructions. Jargon was not associated with understanding diagnosis for patients who had ACS (p = 0.34) or patients who had pneumonia (p = 0.76). Patients with jargon on their discharge instructions were more likely to be white, but otherwise had similar baseline characteristics compared to patients without jargon on their discharge instructions (S2 Appendix).

**Table 2:** Bivariate analysis examining systems predictors to completely understanding hospital diagnosis.

Predictor	Completely understands diagnosis	p value
**Jargon (n = 367)**	** **	0.01
Yes	67 (70.5%)	
No	153 (56.3%)	
**Medical team (n = 363)**		<0.01
Non-teaching	46 (43.4%)	
Housestaff	75 (62.5%)	
Cardiology	97 (70.8%)	
**Outpatient provider aware (n = 328)**		0.63
Yes	168 (59.0%)	
No	22 (55.0%)	
**More than one day notice prior to discharge (n = 374)**		0.71
Yes	111 (60.3%)	
No	111 (58.4%)	

In a multivariate model controlling for age, race, sex, diagnosis, education, income, and medical team, jargon was no longer a significant predictor for completely understanding diagnosis (OR 1.53, 95% CI 0.83–2.82, p = 0.17, see Table 3). However, patients admitted with HF were still less likely to understand their diagnosis (OR 0.37, 95% CI 0.19–0.72, p<0.01).

**Table 3:** Association of jargon and medical team with completely understanding diagnosis (n = 336).

Predictor	Adjusted OR (95% CI)	p-value
**Medical Team (compared to Non-teaching team)**		
Housestaff	2.45 (1.30–4.61)	<0.01
Cardiology	3.83 (1.92–7.63)	<0.01
**Presence of Jargon**	1.53 (0.83–2.82)	0.17

Type of medical team was known for 363 patients. A bivariate analysis demonstrated that housestaff and cardiology team patients were more likely to completely understand their diagnosis (62.5% and 70.8% respectively, see Table 2) compared to non-teaching teams (43.4%, p<0.01). This relationship remained significant in a multivariable analysis (housestaff OR 2.45, 95% CI 1.30–4.62, p<0.01; cardiology 3.83, 95% CI 1.92–7.63, p<0.01). While cardiology teams were more likely to have patients with ACS and less likely to have patients with community-acquired pneumonia, the patients across these different teams had similar baseline characteristics (S3 Appendix).

Data on whether outpatient provider knew whether or not the patient was admitted was available for 328 patients. Outpatient provider being aware of admission was not associated with patient completely understanding diagnosis (59.0% vs 55.0%, p = 0.64).

Data on whether the patient had more than day notice prior to discharge was available on 374 patients. Having more than one day notice prior to discharge was not associated with completely understanding diagnosis (60.3% vs 58.4%, p = 0.71).

## Discussion

In this single site study of patients admitted with ACS, pneumonia, and HF, our analysis found that jargon on discharge instructions, outpatient provider being aware of admission, and having more than one day notice prior to discharge were not associated with completely understanding diagnosis. However, non-teaching team patients were less likely to understand their diagnoses. While previous studies have revealed that a significant portion of patients do not understand their reason for hospitalization [4,6,9] this is the first study, to our knowledge, to explore systems-level, modifiable predictors for understanding reason for hospitalization.

The use of medical jargon has been postulated to worsen physician-patient communication and result in poor patient understanding of their medical condition.[10] This association was demonstrated in a study of diabetes patients by Schillinger et al.[11] However, in our multivariate analysis, we found no association between jargon on discharge instructions and lack of understanding of diagnosis. Schillinger defined jargon based on patient self-report (“How often did your regular doctor use medical words that you did not understand?”) while we defined jargon a priori based on specific terminology. We may have made incorrect assumptions about what type of medical language would be difficult for patients to understand. Specifically, “coronary artery disease” on the discharge instructions was associated with increased understanding, which we did not expect. Moreover, we based our definition of jargon on language in the patient discharge instructions. Patients are educated in many forms, including verbal discussions, and non-discharge instruction education may have contributed to patient understanding despite confusing language in the discharge instructions.

We found non-teaching team patients were less likely to completely understand their reason for hospitalization compared to housestaff and cardiology teams. We hypothesize that patients on housestaff teams may benefit from being rounded on by multiple providers from the same team. This may result in more face-to-face time with the primary team and further reinforcement of the reason for hospitalization. Bedside teaching may also result in collateral education for the patient. There may be other unadjusted factors that may also be contributing, such as the patient’s clinical complexity, cognition, and health literacy. Further work should investigate how these different teams approach patient education.

Our findings appear to be consistent with prior literature on hospitalists that has demonstrated that patients cared for by hospitalists were more likely to have emergency department visits and readmissions after discharge.[12–14] Our study adds to this body of literature, as these prior studies did not investigate factors that may be driving utilization after discharge. We have found that there is decreased understanding of diagnosis on non-teaching teams, and this may be driving this disparate utilization. Further studies should examine whether understanding of diagnosis is linked with utilization.

We investigated outpatient provider knowledge of patient admission, as patients whose outpatient providers knew about their hospitalization may represent those patients whose care was better coordinated, specifically with regards to diagnosis. If both inpatient and outpatient providers communicate to stay “on message” regarding the patient’s reason for hospitalization, this may enhance patients’ ability to understand why they were hospitalized. Outpatient providers who know their patients are hospitalized may also visit or communicate with patients during hospitalization. Patients may feel more comfortable asking their regular outpatient provider questions about their hospital course than their inpatient team. We did not, however, find this to be a significant association with understanding.

Similarly, patients that had more than one day notice prior to discharge may have longer discharge preparation, which could present an opportunity for the patient to have more questions answered about their illness before leaving. However, advance notice was also not associated with better understanding. Our findings highlight the fact that even in cases in which the outpatient provider was aware of hospitalization and there was adequate notice for discharge, many patients still do not understand their reason for admission.

Several patient level factors were noted to be associated with not completely understanding diagnosis. In particular, we found HF diagnosis was associated with not completely understanding diagnosis. We were surprised by this finding because we had hypothesized that patients might better understand chronic diseases, in which they have had more opportunity over time for education. In fact, the patients with HF who did understand their diagnosis appeared to have largely had concomitant ACS, even though the diagnoses written on their discharge instructions were largely classified as medical jargon. It may be that specific diagnoses such as ACS may initiate an institutional-level cascade of patient education unique to that diagnosis, improving patient understanding. ACS patients also may be more likely to be admitted to an intensive care unit. Our analysis did not control for increased level of care during hospitalization. Our findings contrast with a previous study[9] that found cardiac diagnosis was associated with poor understanding of diagnosis. However, this study was conducted in Ireland where the patient population may be different from that in the United States. In addition, that study did not differentiate between ACS and HF.

Lower income and less formal education were other patient level factors that were associated with not completely understanding diagnosis in a bivariate analysis. These socioeconomic factors were not significant in the multivariable analysis, which may reflect that systems-level factors may mitigate these factors with respect to understanding diagnosis. Further studies should investigate which educational interventions are effective in the inpatient setting, and if they can be generalized to different diagnoses.

### Limitations

Our study has several limitations. This is a single site study at an academic center, which limits its generalizability. In addition, our study population was limited to only older patients who were admitted with either ACS, pneumonia, or HF, and thus our findings may not apply to younger patients or those who are admitted with other illnesses. Given that this is an older cohort, in-hospital delirium may affect patients’ ability to understand why they were admitted; however, this study excluded patients who failed a mental status screen. Data on whether the outpatient provider knew if the patient was admitted and if the patient had more than one day notice prior to discharge was obtained from the patient themselves, and may be inaccurate. Our assessment of complete understanding of diagnosis may be considered lenient, and overestimate understanding. In addition, we did not control for the quality of communication patients received. There were an insufficient number of readmitted patients in this study to examine whether these predictors of interest were associated with readmission and we were also underpowered for jargon.

### Conclusions

In summary, this study focused on systems-level, modifiable factors that may impact patients’ ability to understand why they were admitted. We found that non-teaching team patients were less likely to completely understand their diagnosis compared to housestaff and cardiology teams. Heart failure patients were also less likely to understand their reason for admission.

These findings have quality improvement implications as potential targets to reduce readmissions. Further investigation of how provider-patient interaction differs among these groups may aid in the development of effective patient education tools to improve the transition from hospital to community.

## Supporting information

S1 Appendix Definition of intelligible terms for diagnosis section of patient discharge instructions. (DOCX)

S2 Appendix Baseline characteristics comparing patients with jargon on their discharge instructions to patients without jargon on their discharge instructions. (DOCX)

S3 Appendix Baseline characteristics comparing patients on hospitalist, housestaff and cardiology teams. (DOCX)

## References

[1] Jencks SF, Williams MV, Coleman EA. Rehospitalizations among patients in the medicare fee-for-service program. N Engl J Med. 2009;360: 1418–1428. doi: 10.1056/NEJMsa0803563 19339721 10.1056/NEJMsa0803563

[2] Pearson M.L., Mattke S., Shaw R., Ridgely M.S., Wiseman S.H. Patient Self-Management Support Programs: An Evaluation. Final Contract Report (Prepared by RAND Health under Contract No. 282-00-0005) Rockville, MD: Agency for Healthcare Research and Quality; 2007.

[3] Kripalani S, Jackson AT, Schnipper JL, Coleman EA. Promoting effective transitions of care at hospital discharge: a review of key issues for hospitalists. J Hosp Med. 2007;2: 314–323. doi: 10.1002/jhm.228 17935242 10.1002/jhm.228

[4] Horwitz LI, Moriarty JP, Chen C, Fogerty RL, Brewster UC, Kanade S, et al Quality of discharge practices and patient understanding at an academic medical center. JAMA Intern Med. 2013;173: 1715–1722. 1729535 [pii]. doi: 10.1001/jamainternmed.2013.9318 23958851 10.1001/jamainternmed.2013.9318 PMC3836871

[5] Makaryus AN, Friedman EA. Patients' understanding of their treatment plans and diagnosis at discharge. Mayo Clin Proc. 2005;80: 991–994. S0025-6196(11)61579-6 [pii]. doi: 10.4065/80.8.991 16092576 10.4065/80.8.991

[6] Ziaeian B, Araujo KL, Van Ness PH, Horwitz LI. Medication reconciliation accuracy and patient understanding of intended medication changes on hospital discharge. J Gen Intern Med.2012 11;27(11):1513–20. doi: 10.1007/s11606-012-2168-4 Epub 2012 Jul 14. 22798200 10.1007/s11606-012-2168-4 PMC3475816

[7] Horwitz LI, Jenq GY, Brewster UC, Chen C, Kanade S, Van Ness PH, et al Comprehensive quality of discharge summaries at an academic medical center.—J Hosp Med.2013 8;8(8):436–43. doi: 10.1002/jhm.2021 Epub 2013 Mar 22. 23526813 10.1002/jhm.2021 PMC3695055

[8] Sunderland T, Hill JL, Mellow AM, Lawlor BA, Gundersheimer J, Newhouse PA, et al Clock drawing in Alzheimer's disease. A novel measure of dementia severity. J Am Geriatr Soc. 1989;37: 725–729. 2754157 10.1111/j.1532-5415.1989.tb02233.x

[9] Ní Chróinín D. Patient understanding of discharge diagnoses: Prevalence and predictors. European geriatric medicine. 2011;2: 74; 74–78; 78.

[10] Kripalani S, Weiss BD. Teaching about health literacy and clear communication. J Gen Intern Med. 2006;21: 888–890. JGI543 [pii]. doi: 10.1111/j.1525-1497.2006.00543.x 16881953 10.1111/j.1525-1497.2006.00543.x PMC1831575

[11] Schillinger D, Bindman A, Wang F, Stewart A, Piette J. Functional health literacy and the quality of physician-patient communication among diabetes patients. Patient Educ Couns. 2004;52: 315–323. doi: 10.1016/S0738-3991(03)00107-1 14998602 10.1016/S0738-3991(03)00107-1

[12] Kuo YF, Goodwin JS. Association of hospitalist care with medical utilization after discharge: evidence of cost shift from a cohort study. Ann Intern Med. 2011;155: 152–159. doi: 10.7326/0003-4819-155-3-201108020-00005 21810708 10.1059/0003-4819-155-3-201108020-00005 PMC3196599

[13] Go JT, Vaughan-Sarrazin M, Auerbach A, Schnipper J, Wetterneck TB, Gonzalez D, et al Do hospitalists affect clinical outcomes and efficiency for patients with acute upper gastrointestinal hemorrhage (UGIH)? J Hosp Med. 2010;5: 133–139. doi: 10.1002/jhm.612 20235292 10.1002/jhm.612 PMC3587174

[14] Howrey BT, Kuo YF, Goodwin JS. Association of care by hospitalists on discharge destination and 30-day outcomes after acute ischemic stroke. Med Care. 2011;49: 701–707. doi: 10.1097/MLR.0b013e3182166cb6 21765377 10.1097/MLR.0b013e3182166cb6 PMC3304585

