# Supplementary material for: Predictors for patients understanding reason for hospitalization
Source: PLoS One. 2018 Apr 27;13(4):e0196479. doi: 10.1371/journal.pone.0196479 (PMC5922555; doi:10.1371/journal.pone.0196479)
Supplement: S1 Appendix — (DOCX) [file pone.0196479.s001.docx]

| **Diagnosis** | **Intelligible Language** | **Medical Jargon** |
| --- | --- | --- |
| Acute Coronary Syndrome | Heart attack  Cardiac catheterization  Stent  Angioplasty  Chest pain  Chest pressure  Shortness of breath | Unstable angina  Coronary artery disease  CAD  ACS  Acute coronary syndrome  NSTEMI  STEMI  MI  Myocardial infarction  ROMI  PCI  Coronary artery dissection |
| Heart Failure | Heart failure  Congestive heart failure  Fluid in the lungs  Fluid in the legs  Volume overload  CHF  Shortness of breath | Pulmonary edema  Lower extremity edema  Diuresis  SOB  Dilated cardiomyopathy |
| Pneumonia | Pneumonia  Bronchitis  Shortness of breath  Fever | CAP  PNA  Organism NOS  Pulmonary effusion  Respiratory failure  SOB |
| Other | Viral syndrome  Asthma exacerbation  Valvular heart disease  Pacemaker  High INR  COPD exacerbation  Atrial fibrillation  Fast heart rate  Dizziness  Weakness | Pulmonary embolus/emboli  Pulmonary infarct  Intubation  Pleuropericarditis  Atrial flutter  Ventricular tachycardia  Hypertriglyceridemia  Diabetic ketoacidosis  Hyperkalemia  Acute renal failure  Acute kidney injury secondary to dehydration  AV fistula  Angioedema  Septic shock  Cardiogenic shock  Hypovolemic shock  Hypotension  Hypertension  HTN  Bradycardia  Tachycardia  Syncope  Presyncope  Carotid artery stenosis  Aortic aneurysm  MRSA  DM  UTI  Cellulitis  Supratherapeutic INR  Epistaxis  Hemoptysis  Coagulopathy  Acute cholecystitis  Lap cholecystectomy |
