# Supplementary material for: Predictors for patients understanding reason for hospitalization
Source: PLoS One. 2018 Apr 27;13(4):e0196479. doi: 10.1371/journal.pone.0196479 (PMC5922555; doi:10.1371/journal.pone.0196479)
Supplement: S2 Appendix — (DOCX) [file pone.0196479.s002.docx]

| **Characteristic** | **Jargon on discharge instructions (n=95)** | **No Jargon (n=272)** | **p-value** |
| --- | --- | --- | --- |
| **Condition (n=367)** |  |  |  |
| Acute Coronary Syndrome | 77 (81.1%) | 117 (43.0%) | <0.01 |
| Community-Acquired pneumonia | 6 (6.3%) | 82 (30.2%) | <0.01 |
| Heart Failure | 22(23.2%) | 118 (43.4%) | <0.01 |
| **Age, mean (SD) (n=359)** | 76.3 (7.4) | 77.2 (7.5) | 0.30 |
| **Male sex (n=359)** | 59 (63.4%) | 140 (52.6%) | 0.07 |
| **English-speaking (n=356)** | 92 (100%) | 257 (97.3%) | 0.29 |
| **Race/ethnicity (n=359)** |  |  | 0.04 |
| Non-Hispanic white | 85 (91.4%) | 215 (80.8%) |  |
| Non-Hispanic black | 5 (5.4%) | 32 (13.2%) |  |
| Hispanic | 1 (1.1%) | 13 (4.9%) |  |
| Other | 2 (2.2%) | 3 (1.1%) |  |
| **Education (n=354)** |  |  | 0.26 |
| <9th grade | 7 (7.6%) | 33 (12.6%) |  |
| 9th-12th grade | 11 (12.0%) | 44 (16.8%) |  |
| High school diploma or GED | 29 (31.5%) | 71 (27.1%) |  |
| College degree | 33 (35.9%) | 71 (27.1%) |  |
| Graduate degree | 12 (13.0%) | 43 (16.4%) |  |
| **Yearly Income (n=332)** |  |  | 0.51 |
| 0-$18,000 | 22 (24.4%) | 72 (29.8%) |  |
| $18,000-$30,000 | 18 (20.0%) | 33 (13.6%) |  |
| $30,000-$45,000 | 11 (12.2%) | 20 (8.3%) |  |
| $45,000-$65,000 | 5 (5.6%) | 18 (7.4%) |  |
| >$65,000 | 19 (21.1%) | 50 (20.7%) |  |
| No response | 15 (16.7%) | 49 (20.3%) |  |
