# Supplementary material for: Predictors for patients understanding reason for hospitalization
Source: PLoS One. 2018 Apr 27;13(4):e0196479. doi: 10.1371/journal.pone.0196479 (PMC5922555; doi:10.1371/journal.pone.0196479)
Supplement: S3 Appendix — (DOCX) [file pone.0196479.s003.docx]

| **Characteristic** | **Non-Teach (n=106)** | **Housestaff (n=120)** | **Cardiology (n=137)** | **p-value** |
| --- | --- | --- | --- | --- |
| **Condition (n=363)** |  |  |  |  |
| Acute Coronary Syndrome | 29 (27.4%) | 63 (17.4%) | 97 (70.8%) | <0.01 |
| Community-Acquired pneumonia | 49 (46.2%) | 38 (31.7%) | 0 (0%) | <0.01 |
| Heart Failure | 48 (45.3%) | 39 (32.5%) | 54 (39.4%) | 0.08 |
| **Age, mean (SD) (n=356)** | 78.4 (8.3) | 76.1 (7.4) | 76.7 (6.8) | 0.05 |
| **Male sex (n=356)** | 50 (47.6%) | 66 (56.9%) | 80 (59.3%) | 0.18 |
| **English-speaking (n=353)** | 101 (96.2%) | 113 (98.3%) | 131 (98.5%) | 0.31 |
| **Race/ethnicity (n=356)** |  |  |  | 0.15 |
| Non-Hispanic white | 83 (79.1%) | 95 (81.9%) | 118 (87.4%) |  |
| Non-Hispanic black | 14 (13.3%) | 15 (12.9%) | 10 (7.4%) |  |
| Hispanic | 8 (7.6%) | 4 (3.5%) | 3 (2.2%) |  |
| Other | 0 (0%) | 2 (1.7%) | 4 (3.0%) |  |
| **Education (n=350)** |  |  |  | 0.35 |
| <9th grade | 12 (12.1%) | 17 (14.7%) | 11 (8.2%) |  |
| 9th-12th grade | 20 (20.2) | 18 (15.5%) | 15 (11.1%) |  |
| High school diploma or GED | 30 (30.3%) | 31 (26.7%) | 40 (29.6) |  |
| College degree | 27 (27.3%) | 33 (28.5%) | 45 (33.3%) |  |
| Graduate degree | 10 (10.1%) | 17 (14.7%) | 24 (17.8%) |  |
| **Yearly Income (n=328)** |  |  |  | 0.08 |
| 0-$18,000 | 35 (38.9%) | 33 (30.0%) | 28 (21.9%) |  |
| $18,000-$30,000 | 15 (16.7%) | 17 (15.5%) | 19 (14.8%) |  |
| $30,000-$45,000 | 7 (7.8%) | 8 (7.3%) | 15 (11.7%) |  |
| $45,000-$65,000 | 6 (6.7%) | 9 (8.2%) | 8 (6.3%) |  |
| >$65,000 | 8 (8.9%) | 26 (23.6%) | 34 (26.6%) |  |
| No response | 19 (21.1%) | 17 (15.5%) | 24 (18.8%) |  |
